# Association of Circulating Transfer RNA fragments with antibody response to *Mycoplasma bovis* in beef cattle

**DOI:** 10.1186/s12917-018-1418-z

**Published:** 2018-03-13

**Authors:** Eduardo Casas, Guohong Cai, Larry A. Kuehn, Karen B. Register, Tara G. McDaneld, John D. Neill

**Affiliations:** 1 USDA, ARS, National Animal Disease Center, Ames, IA 50010 USA; 2 USDA, ARS, U.S. Meat Animal Research Center, Clay Center, NE 68933 USA

**Keywords:** Cattle, RNA-seq, Selenocysteine, Small non-coding RNA, tRF

## Abstract

**Background:**

High throughput sequencing allows identification of small non-coding RNAs. Transfer RNA Fragments are a class of small non-coding RNAs, and have been identified as being involved in inhibition of gene expression. Given their role, it is possible they may be involved in mediating the infection-induced defense response in the host. Therefore, the objective of this study was to identify 5′ transfer RNA fragments (tRF5s) associated with a serum antibody response to *M. bovis* in beef cattle.

**Results:**

The tRF5s encoding alanine, glutamic acid, glycine, lysine, proline, selenocysteine, threonine, and valine were associated (*P* < 0.05) with antibody response against *M. bovis.* tRF5s encoding alanine, glutamine, glutamic acid, glycine, histidine, lysine, proline, selenocysteine, threonine, and valine were associated (*P* < 0.05) with season, which could be attributed to calf growth. There were interactions (*P* < 0.05) between antibody response to *M. bovis* and season for tRF5 encoding selenocysteine (anticodon UGA), proline (anticodon CGG), and glutamine (anticodon TTG). Selenocysteine is a rarely used amino acid that is incorporated into proteins by the opal stop codon (UGA), and its function is not well understood.

**Conclusions:**

Differential expression of tRF5s was identified between ELISA-positive and negative animals. Production of tRF5s may be associated with a host defense mechanism triggered by bacterial infection, or it may provide some advantage to a pathogen during infection of a host. Further studies are needed to establish if tRF5s could be used as a diagnostic marker of chronic exposure.

## Background

Bovine respiratory disease complex is the most expensive condition in cattle, costing up to $1 billion annually in the United States [1]. Despite the development of vaccines and antibiotics, the condition is responsible for significant morbidity and mortality losses to the cattle industry [2]. Miles [3], suggests that perhaps it is time to look for ways to reduce losses by focusing on the animal’s response to related pathogens, instead of continuing to focus on the pathogens themselves.

*Mycoplasma bovis (M. bovis)* has been identified as a prime pathogen causing respiratory disease of cattle, along with *Pasteurella multocida*, *Mannheimia haemolytica*, and *Histophilus somni* [2, 4, 5]. Common problems with cattle infected with *M. bovis* are chronic sickness, insensitivity to treatment, and inability to reach target weights. *M. bovis* is one of the most common pathogens recovered from lung samples in the abattoir [6].

High throughput sequencing allows identification of small non-coding RNAs [7, 8]. Transfer RNA Fragments (tRFs) are a class of small interfering RNA that were originally considered a degradation product of the translation process, but their role in regulation of gene translation in the cell has now been recognized [9, 10]. Their classification is based on the processing site of the transfer RNA (tRNA): tRFs processed from the 5′ end of the mature tRNA are denoted tRF5; tRFs cleaved at the 3′ end of the mature tRNA are referred to as tRF3; those produced from the beginning of the 3′ end, cleaved from the immature tRNA are designated tRF1 [8, 11, 12]. These tRFs are the second most abundant in tissues, after microRNAs [8]; however, tRFs are the most abundant sncRNAs in serum in cattle, with tRF5s being the predominant group among the three types of tRFs [13].

EN Nolte-‘t Hoen et al. [14], proposed that tRFs are produced in bone marrow and immune cells; however, it has been suggested that other cells may also have the ability to produce them [15, 16]. tRFs have been identified as being involved in inhibition of gene expression in stressed cells and in virus replication [15, 17]. Given their production site and their role in inhibiting gene expression, it is possible they may be involved in mediating the infection-induced defense response [18]. Therefore, our objective was to identify tRF5s associated with serum antibody response to *M. bovis* in beef cattle.

## Methods

### Animals

Bleeding of animals was done according to the management protocol approved by the Animal Care and Use Committee of the Institution. Sera from sixteen beef steers born during spring, 2013, were obtained from the US Meat Animal Research Center, Clay Center Nebraska. Animals were bled at three time points: during summer, 2013, while in the pasture with the dam, at weaning in the fall of the same year, and during summer, 2014. Blood was obtained by jugular venipuncture using a syringe. The samples were centrifuged at 1300 X g for 25 min at 4 °C and serum was aspirated and frozen at − 20 °C until used. Samples were shipped to the National Animal Disease Center, Ames, Iowa.

### Antibody response against *M. bovis*

Cattle sera were tested for antibodies reactive with *M. bovis* using a direct ELISA, as previously reported [19], except that 0.5 μg of antigen was used per well, anti-bovine IgG-peroxidase conjugate (KPL, Inc.), diluted 1:3000 in wash buffer, was used to detect cattle IgG and color development was halted after 45 min. The *M. bovis* isolate M23 was used as the source of antigen [20]. The presence or absence of serum antibody to *M. bovis* was confirmed in each animal using a commercially available ELISA (Biovet, Inc.) prior to selection for inclusion in the appropriate pool. Sera included in the positive pool were 3+ or 4+ positive, on a scale of 1+ to 4+, as described by the ELISA manufacturer. The pool itself tests at 4+ with the Biovet ELISA and has a level of IgG higher than that of the positive control serum provided with the kit. A positive result in our in-house ELISA was defined as an average absorbance at 405 nm greater than the average plus 3 standard deviations of the negative control, calculated independently for each plate analyzed. Sera from the sixteen beef calves collected in summer were ELISA negative for IgG reactive with *M. bovis.* By the fall, eight animals were seropositive (positive group), while eight remained negative (negative group). By spring, all animals in both groups were seropositive.

### tRF isolation

The tRFs were isolated from 200 μl of each serum sample using the miRNeasy Serum/Plasma kit (QIAGEN, Germantown, MD). The tRFs were extracted according to the manufacturer’s direction and the samples were eluted in 14 μl of RNase free water. After extraction 1 μl of each sample was run using the Small RNA chip on an Agilent 2100 Bioanalyzer (Agilent Technologies, Santa Clara, CA), to quantify the tRFs extracted from the samples. The tRFs concentration was determined by using a 10–40 nucleotide gate.

### Library preparation

A sequence library was prepared for each extracted sample. The libraries were prepared using the NEBNext Multiplex Small RNA Library Prep Set for Illumina Set 1 and 2 (New England BioLabs, Ipswich, MA). Each set comprises of 24 unique sequences or barcodes, therefore, 48 unique barcodes were used to identify each sample. Six microliters of each animal’s isolated small RNA fraction was used in library preparation according to manufacturer’s instructions. After the library preparation, libraries were cleaned up and concentrated using the QIAquick PCR purification kit (QIAGEN, Germantown, MD) from 100 μl to a final volume of 27.5 μl. The quality and quantity of the libraries was determined by running 1 μl of each library on a DNA 1000 chip on an Agilent 2100 Bioanalyzer (Agilent Technologies, Santa Clara, CA). The concentration of each indexed library was determined by using a 135–170 nucleotide gate. All the indexed libraries were then pooled and size selected. Five nanograms of each indexed library were used to make the pool, with the total volume of the pool being 246.5 μl. The pool was concentrated using the QIAquick PCR purification kit (QIAGEN, Germantown, MD) to 35 μl of RNase free water. The pool was then size selected using the Pippin Prep on a 3% Agarose gel without added ethidium bromide (SAGE Sciences, Beverly, MA) with a size selection of 142–170 nucleotides according to the manufacturer’s instructions. After the gel was run the pools were concentrated using the QIAquick PCR purification kit (QIAGEN, Germantown, MD) by eluting in 32 μl of RNase free water. One microliter of the size selected library pool was run using a High sensitivity DNA chip Agilent 2100 Bioanalyzer (Agilent Technologies, Santa Clara, CA). The concentration was determined by using a 135–170 nucleotide gate. The final concentration of the size selected pool library was 1.5 nM and the pool was stored at − 20 °C.

### Sequencing the library pool

The pooled size selected library was sequenced using the Hi-Seq Sequencing Kit v2 50 Cycles (Illumina, San Diego, CA) in the Sequencing Core Facility at the National Animal Disease Center (NADC).

### Data analysis

The quality of Illumina sequences was inspected using FastQCv0.11.22 program in the fastx toolkit3. The Illumina adapter was removed using fastx_clipper. Multiple occurrences of unique reads were merged using a custom script. Reads 18–40 bp in size were used in downstream analysis. These reads were first mapped to *Bos taurus* genome (ENSEMBL UMD3.1.75) using Novoalign software (Novocraft Technologies) allowing two mismatches. Those aligned to the genome were then mapped to a database containing different annotated genome features to determine their origin: genomic tRNA sequences were downloaded from http://gtrnadb.ucsc.edu/; mitochondrial tRNA, cDNA, and other non-coding RNA sequences were downloaded from ENSEMBL version 75. The Illumina reads that aligned to tRNA genes or their flanking sequences were further characterized. They were initially aligned to a *Bos taurus* tRNA database using BLASTN and the results were processed using a custom script. Those perfectly aligned to the beginning of mature tRNAs were classified as tRF5. After tRF5 sequences had been determined, their occurrences in Illumina sequences from individual animals were obtained using a custom script. Sequences have been submitted to NCBI Short Read Archive4, under BioProject accession PRJNA319677. Read counts were normalized to library size to reads per million prior to statistical analysis.

### Statistical analysis

Analysis was done using the Mixed procedure of SAS (SAS Inst. Inc., Cary, NC). The model included the effects of ELISA status (positive or negative), season (summer, fall, or spring), and the interaction between ELISA status and season. The present study accounted for the minimum number of samples that could be run and that could provide significant statistical differences. Eight biological replicates (animals) per group suffice the requirements of the study. The power to establish differences at the *P* < 0.05 level, between both groups with a sample size of *n* = 16 is: 1-beta = 0.94.

Probability values shown are nominal and uncorrected for multiple testing. Sixteen animals were used to ascertain the association of tRFs with ELISA status in the present study. Additional experimental units would be needed if significance was adjusted for multiple comparisons. Although next generation sequencing allows profiling tRFs in each experimental unit, the cost associated with embarking on a large scale study is still a limiting factor. The present study was designed to ascertain nominal significant differences with the minimal number of samples. For this reason it was deemed relevant to present un-corrected significances in the present study. Significances should be taken in consideration when interpreting results.

## Results

There were 452,264,204 sequences obtained in the present study. From these, 416,296,523 sequences mapped to tRNA. Only sequences that matched 100% with the tRNA genes and their flanking sequences were included in the study, therefore there were a total of 263,556,821 sequences that matched these criteria. Of these, 261,502,003 sequences were identified as tRF5s. Table 1 shows the number of tRF5s corresponding to the anticodons of each amino acid. The tRF5s with the most number of sequences were tRF5-Glu (*n* = 989,044 + 67,677,835 = 68,666,879), tRF5-Gly (*n* = 156,913,852), tRF5-His (*n* = 23,770,874), tRF5-Lys (*n* = 3,223,746), and tRF5-Val (*n* = 8,103,320). The number of sequences for these five tRF5s comprise 99.7% of the total number of tRF5 sequences in the study. The tRF5s with fewer than 1000 sequences were tRF5-Arg (*n* = 283 + 1 + 42 = 326), tRF5-Ile (*n* = 20), tRF5-Leu (*n* = 165), tRF5-Phe (*n* = 3), tRF5-Ser (*n* = 9), tRF5-Trp (*n* = 11), and tRF5-Tyr (*n* = 4). The remaining tRF5s had more than 1000, but less than 1000,000 sequences.

**Table 1:** Number of 5′-transfer RNA fragments (tRF5) sequences by amino acid and anticodon

Transfer RNA Fragment	Anticodon	Number of sequences
tRF5-Ala	AGC	142,824
tRF5-Ala	CGC	279,970
tRF5-Ala	TGC	2189
tRF5-Arg	CCT	283
tRF5-Arg	TCG	1
tRF5-Arg	TCT	42
tRF5-Asp	GTC	1248
tRF5-Cys	GCA	29,406
tRF5-Gln	CTG	67,818
tRF5-Gln	TTG	25,446
tRF5-Glu	CTC	989,044
tRF5-Glu	TTC	67,677,835
tRF5-Gly	ACC	1
tRF5-Gly	CCC	127,016,832
tRF5-Gly	GCC	29,840,312
tRF5-Gly	TCC	56,707
tRF5-His	GTG	23,770,874
tRF5-Ile	AAT	20
tRF5-Leu	AAG	112
tRF5-Leu	CAG	27
tRF5-Leu	TAG	26
tRF5-Lys	CTT	2,740,190
tRF5-Lys	TTT	483,556
tRF5-Met	CAT	5063
tRF5-Phe	GAA	3
tRF5-Pro	AGG	211,844
tRF5-Pro	CGG	1667
tRF5-Pro	TGG	47,038
tRF5-SelCys	UGA	4579
tRF5-Ser	CGA	4
tRF5-Ser	GCT	1
tRF5-Ser	TGA	4
tRF5-Thr	CGT	16
tRF5-Thr	TGT	3685
tRF5-Trp	CCA	11
tRF5-Tyr	GTA	4
tRF5-Val	AAC	31,138
tRF5-Val	CAC	6,639,535
tRF5-Val	TAC	1,432,647
Total		261,502,003

For identification purposes, the nomenclature of tRF5s will be by amino acid and anticodon. As an example, for the tRF5 for alanine, anticodon AGC, the abbreviation tRF5-AlaAGC will be used.

There were nine tRF5s with different anticodons associated with the outcome of ELISA status (Table 2). The positive group had a greater count of tRF5s when compared to the negative group for all anticodons, with the exception of tRF5-AlaCGC and tRF5-GluCTC. The most significant associations were observed with tRF5-LysCTT (*P* = 0.0002), tRF5-LysTTT (*P* = 0.0057), and with tRF5-SelCys (*P* = 0.002).

**Table 2:** 5′-transfer RNA fragment (tRF5), anticodon, normalized count of tRF5s in serum by group, standard error (SE) and their association (*P*-value) with ELISA status

tRF5	Anticodon	ELISA status		SE	*P*-value
		Negative (RPM)^a^	Positive (RPM)^a^		
tRF5-Ala	CGC	616	494	31	0.0081
tRF5-Glu	CTC	2584	1492	306	0.0155
tRF5-Gly	TCC	65	148	21	0.0082
tRF5-Lys	CTT	3800	6933	534	0.0002
tRF5-Lys	TTT	728	1193	113	0.0057
tRF5-Pro	TGG	78	118	10	0.0093
tRF5-SelCys	UGA	4.9	13.7	1.9	0.0020
tRF5-Thr	TGT	6.4	8.2	0.6	0.0499
tRF5-Val	CAC	11,409	15,104	1275	0.0468

^a^*RPM* Reads per million

Table 3 shows differences in tRF5 counts by season. There were five tRF5s with different anticodon for the same amino acid (tRF5-Ala, tRF5-Gln, tRF5-Gly, tRF5-Pro, and tRF5-Val), associated (*P* = 0.05) with season. For tRF5-AlaAGC, tRF5-AlaTGC, tRF5-ValAAC, tRF5-VAlTAC, tRF5-LysTTT, and tRF5-ProCGG, the greatest number of sequences was observed in summer and fall, 2013, declining by the spring, 2014. For tRF5-GlnTTG, tRF5-GlnTTC, tRF5-GlyTCC, tRF5-SelCysUGA, and tRF5-ThrTGT, the number of copies was the lowest during summer, 2013, consistently increasing throughout the fall, 2013, and spring, 2014. For tRF5-ProAGG, and tRF5-ProTGG, the number of sequences during summer, 2013, and spring, 2014 were the lowest, having the greatest numbers in fall, 2013.

**Table 3:** 5′-transfer RNA fragment (tRF5), anticodon, normalized count of tRF5s in serum by season (summer and fall, 2013, and spring, 2014), standard error (SE) and their association (*P*-value)

tRF5	Anticodon	Season			SE	*P*-value
		Summer, 2013 (RPM)^c^	Fall, 2013 (RPM)^c^	Spring, 2014 (RPM)^c^		
tRF5-Ala	AGC	288^a,b^	386^a^	190^b^	36	0.0017
tRF5-Ala	TGC	4.6^a^	5.9^a^	2.1^b^	0.7	0.0012
tRF5-Gln	CTG	41.6^a^	163.8^b^	196.6^b^	29.9	0.0017
tRF5-Gln	TTG	28.6^a^	53.8^b^	65.9^b^	6.0	0.0003
tRF5-Glu	TTC	161,604^a^	126,622^b^	128,748^b^	8341	0.0074
tRF5-Gly	GCC	47,848^a^	54,304^a^	74,013^b^	5349	0.0035
tRF5-Gly	TCC	65^a^	93^a,b^	161^b^	26	0.0330
tRF5-His	GTG	41,999^a,b^	70,356^a^	35,304^b^	10,090	0.0428
tRF5-Lys	TTT	948^a,b^	1219^a^	714^b^	138	0.0446
tRF5-Pro	AGG	406^a^	562^b^	335^a^	53	0.0124
tRF5-Pro	CGG	4.13^a^	4.14^a^	1.95^b^	0.60	0.0185
tRF5-Pro	TGG	92^a^	138^b^	63^a^	13	0.0006
tRF5-SelCys	UGA	3.9^a^	9.1^a,b^	14.9^b^	2.3	0.0068
tRF5-Thr	TGT	5.7^a^	8.8^b^	7.4^a,b^	0.8	0.0285
tRF5-Val	AAC	68^a^	71^a^	46^b^	7	0.0425
tRF5-Val	TAC	2,666^a,b^	3621^a^	2344^b^	336	0.0277

^a, b^ Means without a common superscript within row are statistically different (*P* < 0.05)

^c^*RPM* Reads per million

**Fig. 1:**
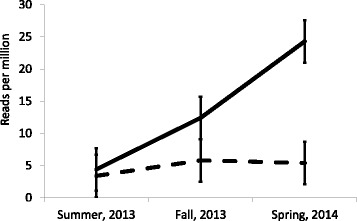
Reads per million by season and ELISA status for tRF-SelCysUGA (*P* = 0.0271). Solid and dashed lines correspond to positive and negative groups, respectively. Animals in the negative group were positive to antibodies against *M. bovis* in the spring, 2014

The interaction for tRF5-SeCys, between ELISA status and season is shown in Fig. 1. During summer, there was no difference in counts for tRF5-SelCysUGA between groups. However, in fall, the positive group had an increased number of sequences, compared to the negative group. The difference in number of sequences of tRF5-SelCysUGA between the positive and negative groups increased further during spring, 2014. In Mycoplasmas the codon UGA, typically a stop codon, is instead translated as tryptophan [21]. It was determined that tRF5-SelCys identified in the present study were of bovine origin.

**Fig. 2:**
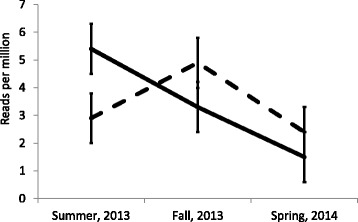
Reads per million by season and ELISA status for tRF-ProCGG (*P* = 0.0489). Solid and dashed lines correspond to positive and negative groups, respectively. Animals in the negative group were positive to antibodies against *M. bovis* in the spring, 2014

Figure 2 shows the interaction for tRF5-ProCGG, between ELISA status and season. During summer, 2013, both groups have similar number of sequences. A steady decline in the number of sequences was observed for the positive group throughout fall, 2013, and spring, 2014. For the negative group, the number of sequences increased from summer to fall. In spring, the negative group has a decline of tRF5-ProCGG was also detected.

**Fig. 3:**
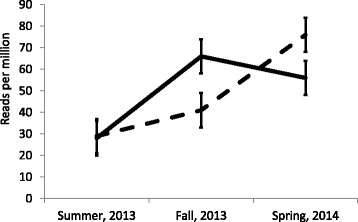
Reads per million by season and ELISA status for tRF5-GlnTTG (*P* = 0.0406). Solid and dashed lines correspond to positive and negative groups, respectively. Animals in the negative group were positive to antibodies against *M. bovis* in the spring, 2014

A different pattern was observed for tRF5-GlnTTG (Fig. 3). There was no difference in the number of sequences between groups in summer, 2013. In fall, the positive group had a greater number of sequences when compared to the negative group. In spring, 2013, there was no difference in the number of sequences between groups. The slope of the increase from summer to fall in the positive group, and from fall to spring in the negative group was similar.

## Discussion

It has been indicated that tRFs, tRF5s specifically, may be a significant regulator of gene silencing when animals are faced with pathogens [17]. There is a limited number of studies that have evaluated tRFs in species other than cattle [7, 11, 22], and a single study that has evaluated tRF response to a virus in cell culture [17]. Production of tRFs has been observed when cells are under stress, regardless of it being physical, chemical, or by a viral infection [15, 17, 18]. It has been established that under normal conditions, tRF5s are the predominant molecules in serum, compared to tRF3s and tRF1s [13]. Therefore, the present study characterizes the tRF5 response in cattle to exposure to the respiratory pathogen *M. bovis*.

In the present study, tRF5s for selenocysteine were identified as being differentially expressed between groups positive or negative with a *M. bovis* ELISA, throughout the growth period of the animals (Fig. 1). Selenocysteine has been recognized as the 21st amino acid [23]. It has been established that selenoproteins, which comprise selenocysteine, are produced by prokaryotic and eukaryotic organisms [24, 25]. It has been shown that pectoral muscles of poultry express selenoproteins, which makes them a primary target of selenium deficiency diseases [26]. It has also been established that selenoproteins in macrophages protect mice from dextran sodium sulfate colitis [27]. However, the biological implication of structural differences between transfer RNA and selenocysteine transfer RNA still remains to be fully understood [28]. Figure 1 shows a greater number of tRF5-SelCysUGA in the group that became exposed to the pathogen between summer and fall, 2013, with the number increasing from fall to spring in the same group. However, although the negative group was negative in the fall, 2013, and became positive by the spring, 2014, no increase in the counts of tRF5-SelCysUGA was observed in the latter group. This could indicate that chronically exposed animals are prone to increase counts of tRF5-SelCysUGA. Selenocysteine is a rarely used amino acid that is incorporated into proteins by the opal codon (UGA). Production of tRF5-SelCysUGA may be associated with a host defense mechanism triggered by the bacterial infection; however, it is also possible that it may provide some advantage to the pathogen during the infection of the host. Further studies would be needed to ascertain if the tRF5-SelCysUGA could be used as a diagnostic indicator of chronic exposure.

The number of sequences for tRF5-ProCGG, declined as animals became ELISA positive (Fig. 2). A proline-rich peptide (PRP), isolated from the bovine neurohypophysis, has been shown to regulate immune activity, preventing death of mice infected with Gram-negative bacteria [29]. The PRP has a regulatory role in the oxidative burst induction of normal and relapsing inflammatory diseases cells such as neutrophils and monocytes [30, 31]. *Mycoplasma bovis* is a Gram-negative bacterium that could initiate or disrupt the regulation of PRP in infected cattle. Diminishing counts of tRF5-ProCGG may be a sign the host is producing additional mature transfer RNA for proline to generate additional PRP molecules to defend against *M. bovis*, thus, depleting the circulating amounts of this tRF5. However, it is also possible that diminishing counts of tRF5-ProCGG in ELISA positive animals to *M. bovis* could be a defense mechanism from the bacterium trying to inhibit the production of PRP in the host. Additional studies need to be developed to fully understand the role of this tRF5 in the defense mechanism of the host.

The counts of tRF5-GlnTTG increased as animals became ELISA positive (Fig. 3). There was a similar response in the positive and negative groups. The positive group increased the number of sequences of tRF5-GlnTTG between summer and fall, 2013; whereas the negative group increased it between fall, 2013, and spring, 2014. A review of the effect of administration of glutamine in acute respiratory disease syndrome in humans suggests that it reduces lung inflammation and mortality, while increasing alveolar barriers and oxygenation [32]. GP Oliveira, MG de Abreu, P Pelosi and PR Rocco [32], indicate that administration of exogenous glutamine may be beneficial in respiratory disease, representing a potential therapeutic tool for the condition. The association of number of sequences of tRF5-GluTTG could be associated with the use of glutamine by the host in response to a *M. bovis* infection.

When Tables 2 and 3 are compared, it is recognized that tRF5-Ala and tRF5-Val have a distinctive pattern. For tRF5s of both amino acids, two of the three anticodons are associated with season, while the third anticodon (tRF5-AlaCGC and tRF5-ValCAC) is associated with antibody response to *M. bovis.* This pattern can also be observed between tRF5-GluCTC (associated with antibody response to *M. bovis*), and tRF5-GluTTC (associated with season). This comparison shows that one of the tRF5s for each amino acid is not modified by season. These tRF5s could be likely targets as diagnostic indicators of exposure to *M. bovis.*

The ELISA used here to identify cattle as positive or negative for *M. bovis* is based on the reactivity of serum IgG with an extract enriched in membrane proteins of the bacterium. ELISAs of similar design are commonly used for detection of *M. bovis*-infected animals in both research and diagnostic settings. However, it is not known whether cross-reactive antibodies that may be elicited by infection with related commensal species, such as *M. bovirhinis*, could falsely contribute to estimates of *M. bovis*-specific antibody in individual animals, including those in this study. Nonetheless, seroconversion on a group level is predictive of *M. bovis* infection, especially when antibody titers are high [33], as we found here for sera from animals in the positive group. At the fall, 2013 sampling, when positive and negative groups were defined, the level of serum antibody in all 8 positive animals exceeded the level in the pool of sera used as a positive control, in most cases by at least two-fold. Therefore, we expect any cross-reactive antibodies detected by the ELISA to have had a negligible effect on the accurate identification of cattle infected with *M. bovis*.

Growth of the animal could be responsible for expression of tRF5s in different seasons. There have been no studies involving the expression of tRF5s in growing cattle. Expression of other small non-coding RNAs such as microRNAs has been compared between muscle tissue from fetal and adult cattle [34, 35], but there are no studies evaluating tRF5s. There were seven tRF5s (tRF5-AlaAGC, tRF5-AlaTGC, tRF5-HisGTG, tRF5-LysTTT, tRF5-ProCGG, tRF5-ValAAC, and tRF5-ValTAC), that were down-regulated in spring, 2014, when compared to summer, and fall, 2013. These tRF5s could be essential for development of the calves at initial stages of growth, and their relevance diminishes as the animal reaches later physiological stages (i.e. puberty). There were other group of tRF5s that had the opposite profile. These tRF5s (tRF5-GlnCTG, tRF5-GlnTTG, tRF5-GlyTCC, tRF5-SelCysUGA, and tRF5-ThrTGT), were down-regulated in summer, 2013, when compared to fall, 2013, and spring, 2014. This group of tRF5s could be relevant as the animal reaches latter stages, while being unimportant during initial phases of growth. Two tRF5s (tRF5-ProAGG and tRF5-ProTGG) were upregulated only during fall, 2013. It is possible these tRF5s are related to weaning because their up-regulation coincided with the age at which calves are naturally weaned from the mother. tRF5-GluTTC and tRF5-GlyGCC were upregulated in summer, 2013, and in spring, 2014, respectively. It is unknown if their importance is due to their upregulation during these seasons or the growth stage at which the animals are in. Additional studies should be developed to address this question. Animals in this study were born in spring, 2013, and raised under semi-commercial conditions until reaching slaughter weight, at approximately 1.25 years of age. Given there is no prior information of tRF5 production in growing beef cattle, it can only be assumed that growth is responsible for differences in tRF5 production by season.

The tRF5s with the most sequences were tRF5-Glu, tRF5-Gly, tRF5-His, tRF5-Lys, and tRF5-Val, while the tRF5s with the fewest counts were tRF5-Arg, tRF5-Ile, tRF5-Leu, tRF5-Phe, tRF5-Ser, tRF5-Trp, and tRF5-Tyr. This is a similar pattern previously observed [13]. Casas et al. [13], used mature dairy cows to establish the proportion of tRF5s for each amino acid, while the present study comprises growing crossbred beef cattle. Given that proportions of each tRF5 are similar between both studies, it is likely the proportions of tRF5s for each amino acid observed are characteristic of the species.

Diagnosis of exposure to pathogens is important in cattle production. There is a need to identify biomarkers for early diagnosis of disease. Using small non-coding RNAs as diagnostic markers has been proposed as an alternative to traditional methods [36, 37]. Serum is a readily available biological sample, and it has been established that serum small non-coding RNAs such as microRNAs are stable even after being exposed to conditions that degrade RNA [38]. Transfer RNA Fragments are a novel group of small non-coding RNAs that circulate in serum. These molecules may even be a better candidate for use as a biomarker given their abundance in serum of cattle [13].

## Conclusions

We identified nine tRF5s for which expression levels were associated with ELISA status*.* There were tRF5s associated with differences in expression due to season, which could be attributed to calf growth. A selenocysteine tRF5 was also identified as differentially expressed between animals positive and negative to a *M. bovis* ELISA. It is unclear the role of this tRF5, but it is known that selenocysteine plays an important role in the defense mechanism of the host when colonized by a pathogen. These data suggest that the selenocysteine tRF5 could be a potential biomarker to identify cattle exposed to *M. bovis.* 5′ transfer RNA fragments that were differentially expressed by ELISA status could also be considered as potential biomarkers. Further studies should be conducted to establish if these transfer RNA fragments could be used as a diagnostic indicator of exposure to *Mycoplasma bovis*.

## Abbreviations

Ala: Alanine
Arg: Arginine
Asp: Aspartic acid
Cys: Cysteine
ELISA: Enzyme-linked Immunosorbent Assay
Gln: Glutamine
Glu: Glutamic acid
Gly: Glycine
His: Histidine
IgG: Immunoglobulin G
Ile: Isoleucine
Leu: Leucine
Lys: Lysine
*M. bovis*: *Mycoplasma bovis*
Met: Methionine
Phe: Phenylalanine
Pro: Proline
RNA: Ribonucleic acid
SelCys: Selenocysteine
Ser: Serine
Thr: Threonine
tRF5s: 5′ transfer RNA fragments
tRFs: Transfer RNA Fragments
tRNA: Transfer RNA
Trp: Tryptophan
Tyr: Tyrosine
Val: Valine
